# Dr. Virgil O. Hardon

**Published:** 1904-04

**Authors:** 


Dr. Virgil O. Hardon, of Atlanta, died of pneumonia at his home in that city, February 7, 1904, aged 54 years. Dr. Hardon was a physician of repute and achieved fame in the practice of gynecology. He was professor of gynecology and obstetrics in the College of Physicians and Surgeons at the time of his death, and his colleagues testified their respect for his memory by acting as pallbearers at the obsequies. The Atlanta Academy of Medicine also formed an honorary escort at the funeral. Dr. Hardon was a man of genial presence, of exceptional humor, and a royal companion in the social circle. He was, withal, an excellent teacher as well as a skilful physician. The *Atlanta Journal-Record of Medicine* for March, 1904, devotes its leading editorial page to a touching tribute to the memory of Dr. Hardon.

